# Supplementary material for: An antibody against L1 cell adhesion molecule inhibits cardiotoxicity by regulating persistent DNA damage
Source: Nat Commun. 2021 Jun 2;12:3279. doi: 10.1038/s41467-021-23478-1 (PMC8172563; doi:10.1038/s41467-021-23478-1)
Supplement: Supplementary file 1 — Supplementary Information [file 41467_2021_23478_MOESM1_ESM.pdf]

Supplementary Figure 1

a

Intensity	Symbol	Name
2.14	L1CAM	L1 cell adhesion molecule

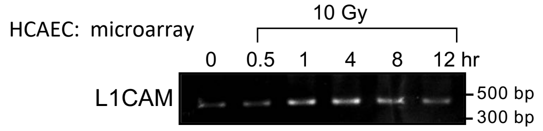

b

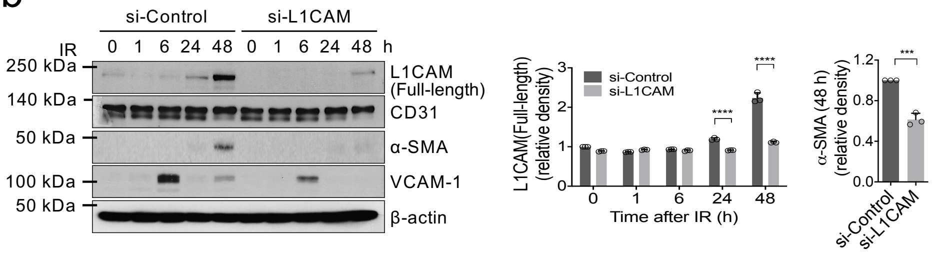

c

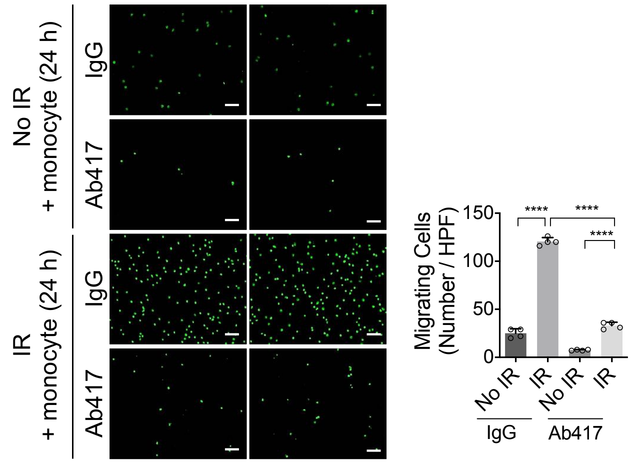

Supplementary Fig. 1. L1CAM knockdown or anti-L1CAM antibody treatment inhibits irradiation (IR)-induced endothelial damage. (**a**) HCAECs were harvested 12 h after IR (10 Gy) and subjected to microarray analysis. Microarray data and RT-PCR results of L1CAM are shown 12 h after IR (10 Gy) (**b**) Immunoblotting for full-length L1CAM, CD31, α-SMA, VCAM-1, and β-actin in L1CAM-knockdown HUVECs at the indicated time points after irradiation (10 Gy). Quantification of full-length L1CAM (n=4 independent experiments, **** p<0.0001, two-way ANOVA) and α-SMA (n=3 independent experiments, p=0.0004, Student's t-test) is shown. Error bars represent mean ± SD from independent experiments. **(c)** Monocyte transmigration assay using irradiated HUVECs pretreated with or without Ab417. HUVECs were pretreated with control IgG or Ab417, irradiated (10 Gy), and co-cultured with Green 5-chloromethylfluorescein diacetate (CMFDA)-labeled monocytes for 24 h. Media from the lower chamber containing migrated monocytes were imaged (left panel). Migrated monocytes were counted (right panel). Scale bar = 20 µm. Quantification of migrating cells number per field is shown (magnification, 100×; n=4). Error bars represent mean ± SD (**** : p<0.0001, one-way ANOVA for multiple comparisons). Data are representative of three independent experiments. IR, irradiation.

Supplementary Figure 2

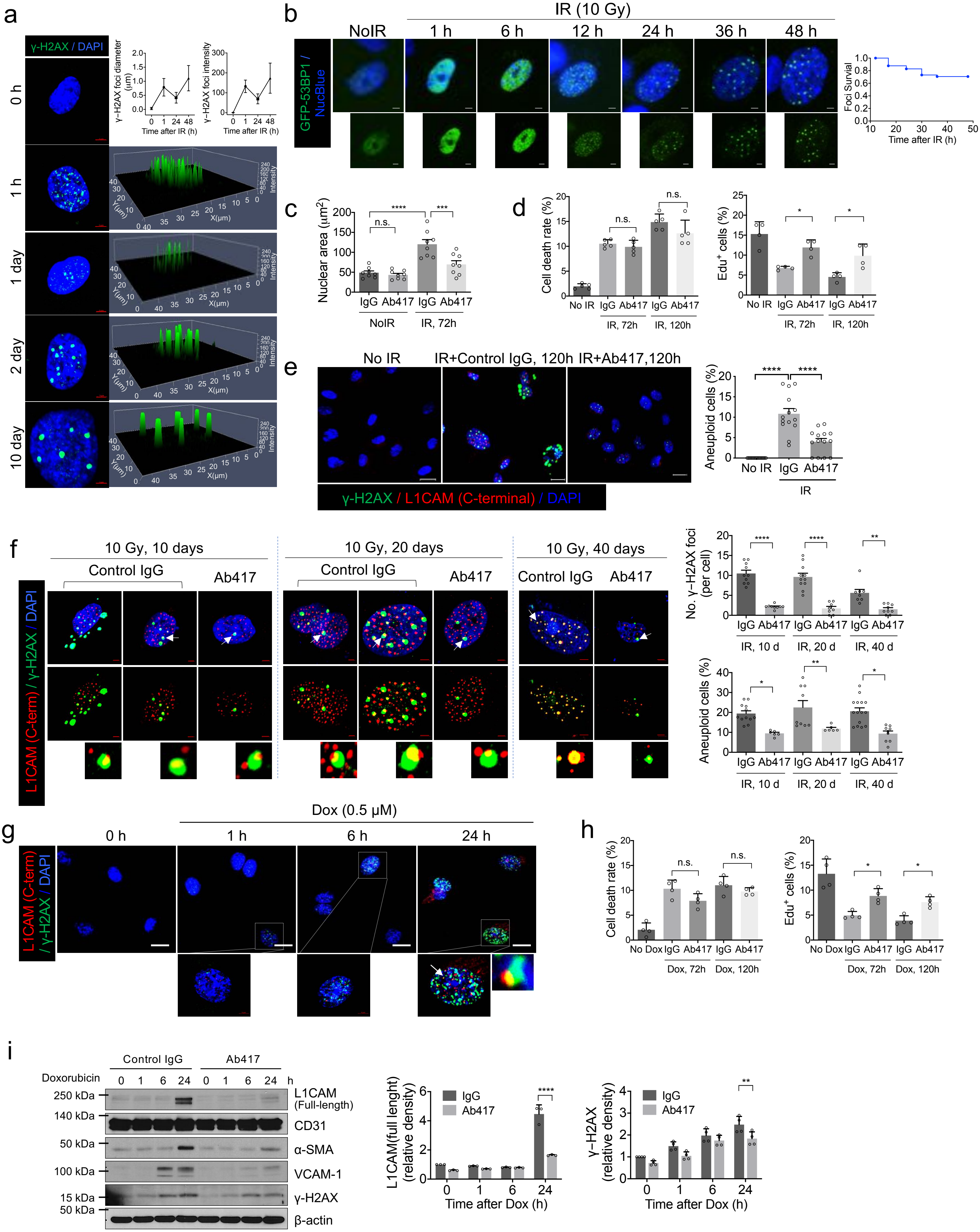

Supplementary Fig. 2. L1-CT fragments colocalize with persistent γ-H2AX foci after irradiation (IR) or doxorubicin (Dox) treatment in HUVECs. (**a**) Immunofluorescence staining of γ-H2AX in HUVECs 1 h, and 1, 2, and 10 days post IR (10 Gy) (magnification, 400×). Scale bar = 5 μm. Quantification of the intensity and diameter of γ-H2AX foci from 3D micrographs (right). For quantification of the intensity and diameter of γ-H2AX foci were counted at least 10 cells per field (magnification, 400×, *n*=9). (**b**) HUVECs were transfected with the GFP-53BP1 vector. Representative time-lapse images of HUVECs expressing GFP-53BP1 after IR at the indicated times are shown. Kaplan-Meier survival curves for GFP-53BP1 foci 12 h after 10 Gy IR at the indicated times. Ab417 pretreat >300 foci from 10 cells were tracked at the indicated times (P < 0.001). **(c)** Cell nuclear area in HUVECs 72h post IR (10 Gy) with control IgG or ment. For quantification of cell nuclear area were counted at least 10 cells per field. (magnification, 400×, *n*=8). Error bars represent mean ±SEM (No IR+IgG vs No IR+Ab417 *p*=0.9665; No IR+IgG vs IR+IgG *p*<0.0001; IR+IgG vs IR+Ab417 *p*=0.0007). **(d)** Cell death rate in HUVECs 72 and 120 h post IR (10 Gy) with control IgG or Ab417 pretreatment determined via FACS analysis using propidium iodide (*n*=5 biologically independent experiments). Edu^+ cell percentage in HUVECs 72 and 120 h post IR (10 Gy) with control IgG or Ab417 pretreatment determined via FACS analysis using Edu staining (*n*=4 independent experiments, IR72h IgG vs Ab417 *p*=0.0267; 120h IgG vs Ab417 *p*=0.0219). Error bars represent mean ±SD from independent experiments. **(e)** Immunofluorescence staining for γ-H2AX and L1-CT in HUVECs 120 h post IR (10 Gy) with control IgG or Ab417 pretreatment (magnification, 400×). Quantification of aneuploid cells is shown. Scale bar = 10 μm. Aneuploid cells were counted at least 10 cells per field (magnification, 400×, *n*=14). Error bars represent mean ±SEM (**** : *p*<0.0001). **(f)** Immunofluorescence staining for γ-H2AX and L1-CT in HUVECs 10, 20, 40 days post IR (10 Gy) with control IgG or Ab417 pretreatment (magnification, 400×). Quantification of aneuploid cells and γ-H2AX foci is shown. Scale bar = 5 μm. Aneuploid cells and γ-H2AX foci in each sample were counted at least 10 cells per field (magnification, 400×, *n*≥6). Error bars represent mean ±SEM (Aneuploid cells IR 10d *p*=0.0201; IR 20d *p*=0.002; IR 40d *p*=0.0007, γ-H2AX foci IR 10d *p*<0.0001; IR 20d *p*<0.0001; IR 40d *p*=0.0034). **(g)** Immunofluorescence staining for γ-H2AX and L1-CT in HUVECs 1, 6, and 24 h post Dox treatment with control IgG or Ab417 pretreatment (magnification, 400×). Scale bar = 20 μm (enlarged, 5 μm). **(h)** The cell death rate in HUVECs 72 h after Dox treatment with control IgG or Ab417 pretreatment determined via FACS analysis using propidium iodide (*n*=4 independent experiments). Edu^+ cell percentage in HUVECs 72 h after Dox treatment with control IgG or Ab417 pretreatment determined via FACS analysis using Edu staining (*n*=4 independent experiments, IR72h IgG vs Ab417 *p*=0.0305; 120h IgG vs Ab417 *p*=0.0427). Error bars represent mean ±SD from independent experiments. **(i)** Immunoblotting of full-length L1CAM, CD31, α-SMA, VCAM-1, γ-H2AX, and β-actin in HUVECs 0, 1, 6, and 24 h after Dox treatment and quantification of full-length L1CAM (*n*=4 independent experiments, 24h *p*<0.0001) and γ-H2AX (*n*=3 independent experiments, 24h *p*=0.0039). Error bars represent mean ±SD from independent experiments. ns: not significant. (**c, d, e, f, h :** one-way ANOVA for multiple comparisons, **i :** two-way ANOVA for multiple comparisons)

Supplementary Figure 3

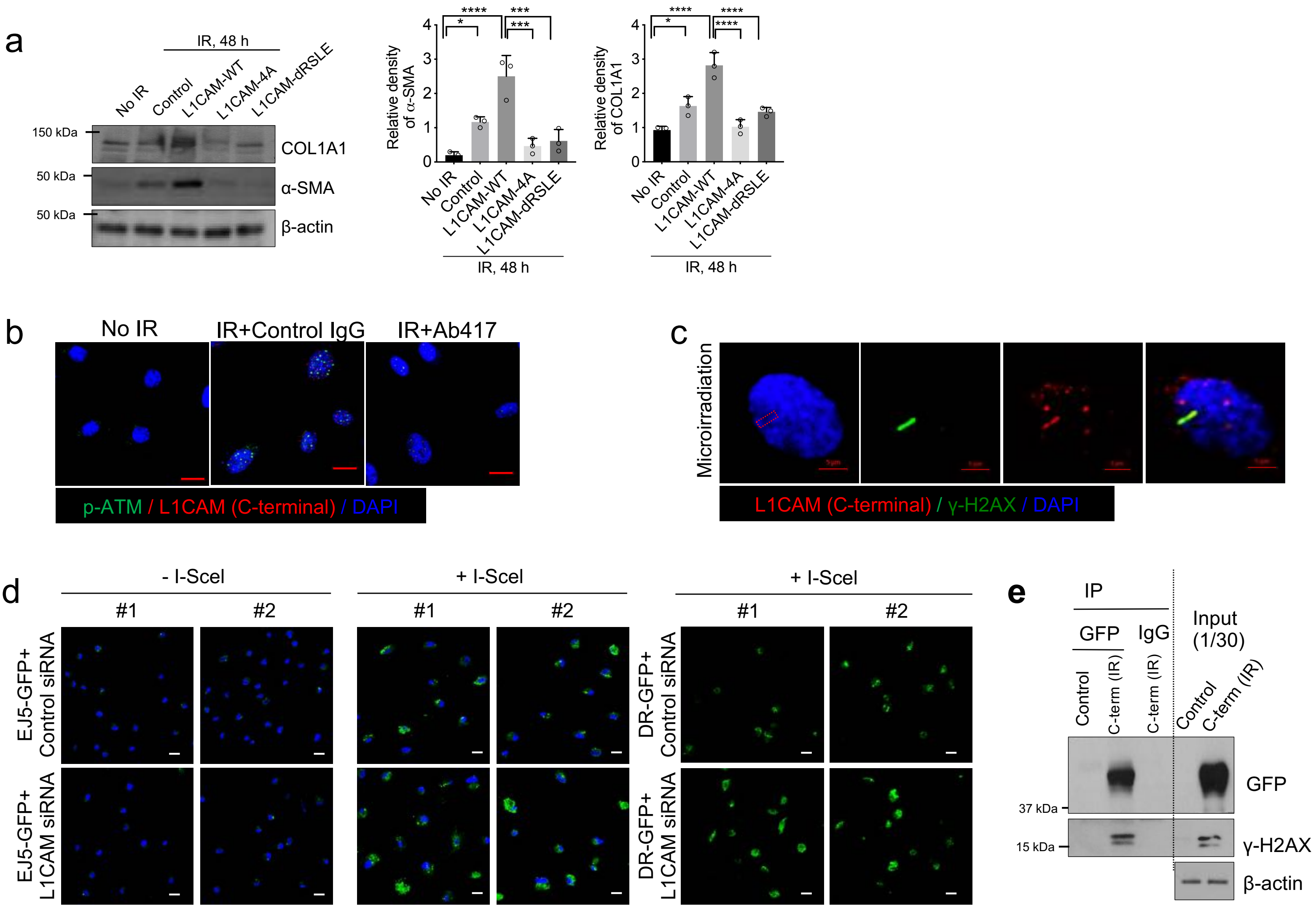

Supplementary Fig. 3 L1CAM deletion increases DNA repair in HUVECs. (a) HUVECs were transfected with human full-length (L1-WT), NLS-mutated (L1-4A), and endocytosis-deficient (L1-dRSLE) L1CAM vectors after knockdown of endogenous L1CAM. Immunoblotting of COL1A1, α-SMA, and β-actin. Quantification of COL1A1 (No IR vs Control+IR p=0.0311; **** : p<0.0001) and α-SMA (No IR vs Control+IR p=0.0353; No IR vs L1CAM-WT+IR p<0.0001; L1CAM-WT+IR vs L1CAM-4A+IR p=0.002; L1CAM-4A+IR vs L1CAM-dRSLE+IR p=0.0003) in HUVECs 48 h post irradiation (IR) (10 Gy). Error bars represent mean ±SD from n=3 independent experiments (one-way ANOVA for multiple comparisons). **(b)** Immunofluorescence staining for p-ATM and L1-CT in HUVECs 48 h post IR (10 Gy) with control IgG or Ab417 pretreatment (magnification, 400×). Scale bar = 20 µm. **(c)** Immunofluorescence staining for L1-CT and γ-H2AX in HUVECs 15 min after microirradiation. Scale bar = 5 µm. **(d)** Immunofluorescence detection of GFP^{+} cells resulting from DNA repair in L1CAM-knockdown HUVECs (magnification, 400×). Scale bar = 20 µm. HUVECs were transiently transfected with DR-GFP or EJ5-GFP constructs with control or L1CAM siRNA, followed by pBAD-I-SceI transfection to induce DNA damage. (e) HUVECs were transfected with L1-CT lentiviral vectors tagged with N-terminal GFP. Immunoblotting of γ-H2AX after GFP immunoprecipitation in HUVECs expressing L1-CT 72 h post IR (10 Gy).

Supplementary Figure 4

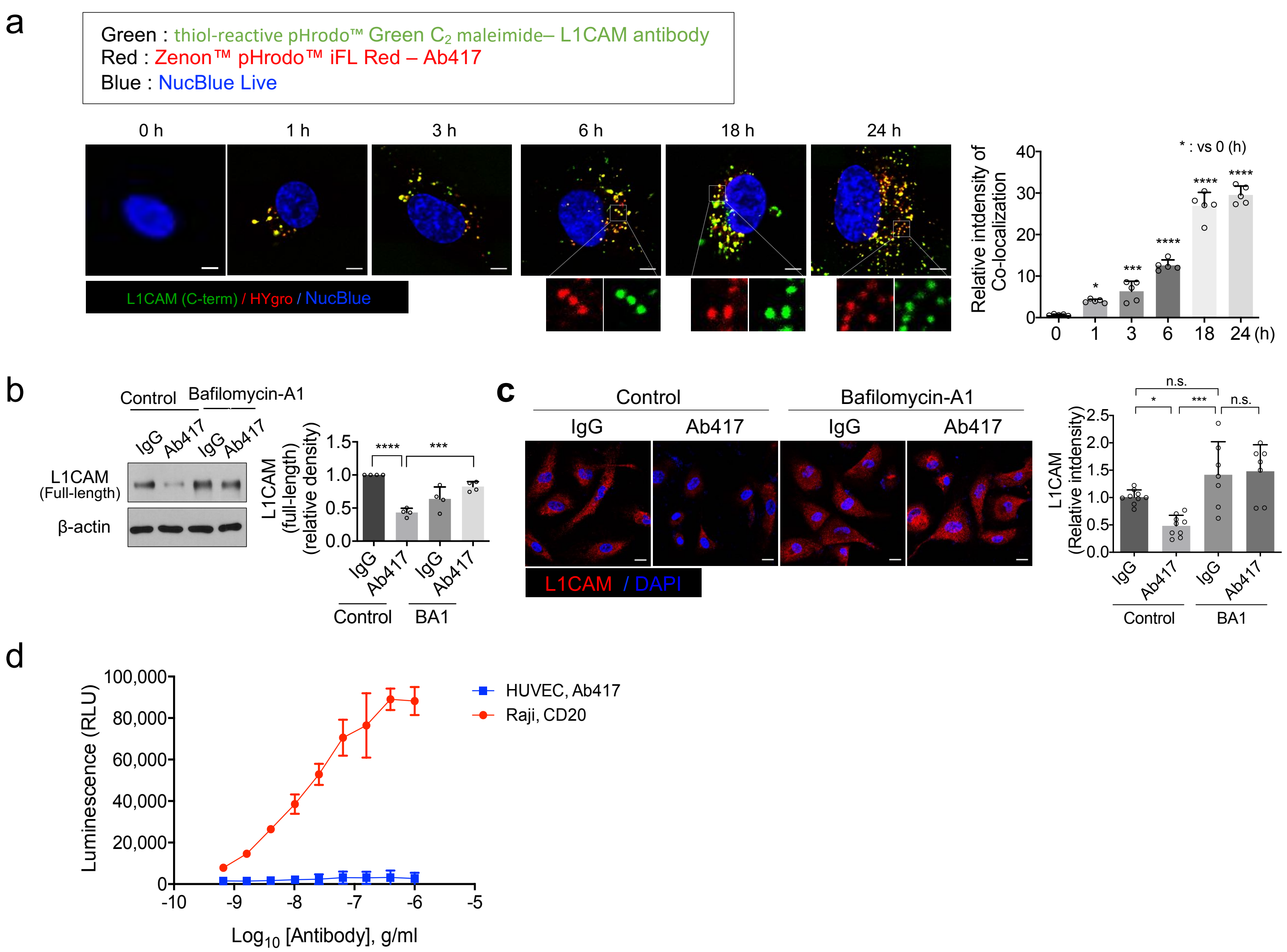

Supplementary Fig. 4. Internalization of L1CAM and Ab417 in HUVECs. (a) pHrodo™ Green-labeled L1CAM and Zenon™ pHrodo™ iFL red-labeled Ab417. Time-lapse of HUVECs with L1CAM (green) and Ab417 (red) at the indicated times using confocal microscopy. Nuclei were stained with NucBlue Live (blue) (magnification, 400×). Scale bar = 5 μm. 33342. Quantification of the co-localization of L1CAM (green) and Ab417 (red) per field is shown (magnification, 400×; n=5). Error bars represent mean ±SD (0h vs 1h p=0.0493; 0h vs 3h p=0.0005; **** p<0.0001). **(b)** HUVECs were transfected with L1-WT lentiviral vector. Immunoblotting for full-length L1CAM and β-actin 24h after control IgG or Ab417 treatment in HUVECs expressing L1-WT with/without pretreatment of Bafilomycin-A1 (100 nM). The quantification of full-length L1CAM expression. Error bars represent mean ±SD from n=3 independent experiments (Control+IgG vs Control+Ab417 p<0.0001; Control+Ab417 vs BA1+Ab417 p=0.0009). **(c)** Immunofluorescence staining for L1CAM 24h after control IgG or Ab417 treatment in HUVECs expressing L1-WT pretreated with Bafilomycin-A1. Quantification of full-length L1CAM intensity per field is shown (magnification, 400×, n≥7). Scale bar = 20 μm. Error bars represent mean ±SD (Control, IgG vs Control, Ab417 : p=0.0309, Control, Ab417 vs BA1, IgG : p=0.0002). (d) ADCC Bioassay Target Cells (Raji, HUVEC) were respectively incubated with a series of concentrations of antibody (Anti-CD20, Ab417) followed by the addition of ADCC Bioassay Effector Cells. Error bars represent mean ±SD from n=3 biologically independent samples. (**a,b,c,d** : one-way ANOVA for multiple comparisons).

Supplementary Figure 5

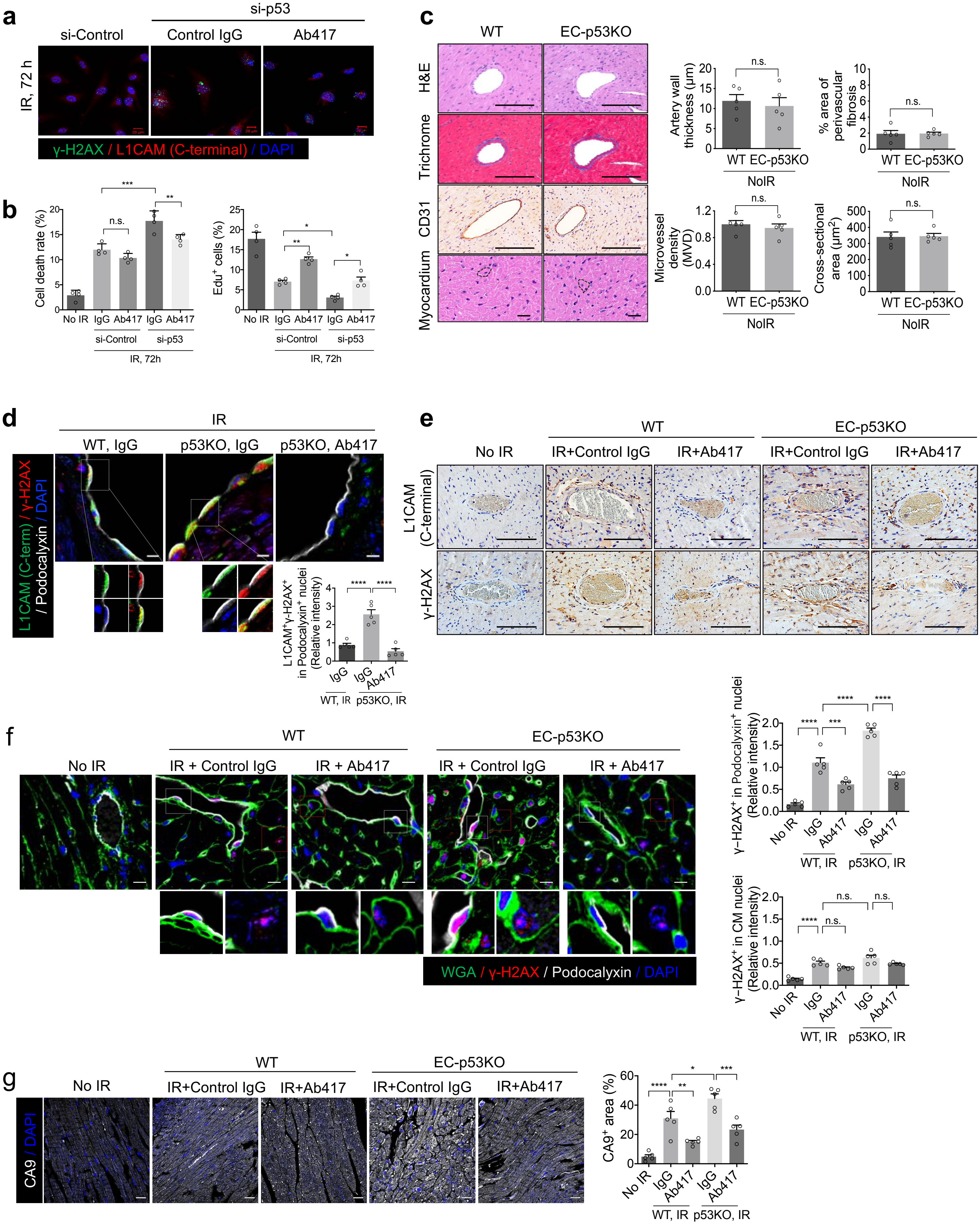

Supplementary Fig. 5 Anti-L1CAM antibody prevents p53 deficiency-induced increase in L1CAM C-terminal (L1-CT) expression and γ-H2AX foci formation (a) Immunofluorescence staining of γ-H2AX and L1-CT in HUVECs 72 h post 10-Gy IR. HUVECs were transfected with p53 siRNA and pretreated with control IgG or Ab417 (20 μg/mL) before irradiation (IR). (**b**) Cell death rate using propidium iodide and Edu^+ cell percentage in HUVECs 72 h post IR (10 Gy) via FACS analysis. Cells were treated with p53 siRNA and pretreated with control IgG or Ab417 (20 μg/mL) before IR with control IgG or Ab417 pretreatment, then detected via FACS analysis. Error bars represent mean ±SD from n=4 biologically independent experiments **(**Cell death rate : si-Control+IgG vs si-p53+IgG p=0.0001; si-p53+IgG vs si-p53+Ab417 p=0.007, Edu^+ cell percentage : si-Control+IgG vs si-Control+Ab417 p=0.0089; si-Control+IgG vs si-p53+IgG p=0.0478; si-p53+IgG vs si-p53+Ab417 p=0.0318). **(c)** Hematoxylin and eosin staining, Masson's trichrome staining, and immunohistochemical detection of CD31 in the cardiac tissue of wild-type or endothelial cell-specific *Trp53* knockout (EC-p53KO) mice (n=5 animals per group). Quantification of arterial wall thickness, perivascular fibrosis area, microvessel density (magnification, 200×, Scale bar = 100 μm), and myocyte cross-sectional area per field (magnification, 400×, Scale bar = 20 μm). Error bars represent mean ±SEM. (**d**) Immunofluorescence detection of colocalization of L1-CT and γ-H2AX in cardiac tissues 3 weeks post IR. Podocalyxin was used as an EC marker (magnification, 400×). Scale bar = 5 μm. Wild-type or EC-p53KO mice were injected intravenously with control IgG or Ab417 (10 mg/kg) three times a week and received 17.5-Gy thoracic IR (n = 5 animals per group). Scale bar = 20 μm. Error bars represent mean ±SEM (**** : p<0.0001). (**e**) Immunohistochemical detection of L1-CT and γ-H2AX in cardiac tissues 3 weeks post IR (magnification, 200×). Scale bar = 100 μm. **(f)** Immunofluorescence staining of WGA, γ-H2AX, and podocalyxin (as an EC marker). Scale bar = 10 μm (magnification, 400×). Quantification of nuclear γ-$H2AX^+$ cells among $podocalyxin^+$ cells. Error bars represent mean ±SEM of n=5 animals per group. (WT IgG vs Ab417 : p=0.0006, **** : p<0.0001). **(g)** Immunofluorescence staining of CA9 and quantification of $CA9^+$ area per field (magnification, 400×). Scale bar = 20 μm. Error bars represent mean ± SEM from n=*5* animals per group (No IR vs WT IR+IgG p<0.0001; . WT IR+IgG vs WT IR+Ab417 p=0.0086; WT IR+IgG vs p53KO IR+IgG p=0.0313; p53KO IR+IgG vs p53KO IR+Ab417 p=0.0006). (**c** : Student's *t*-test, **b, e, f, g** : one-way ANOVA for multiple comparisons).

Supplementary Figure 6

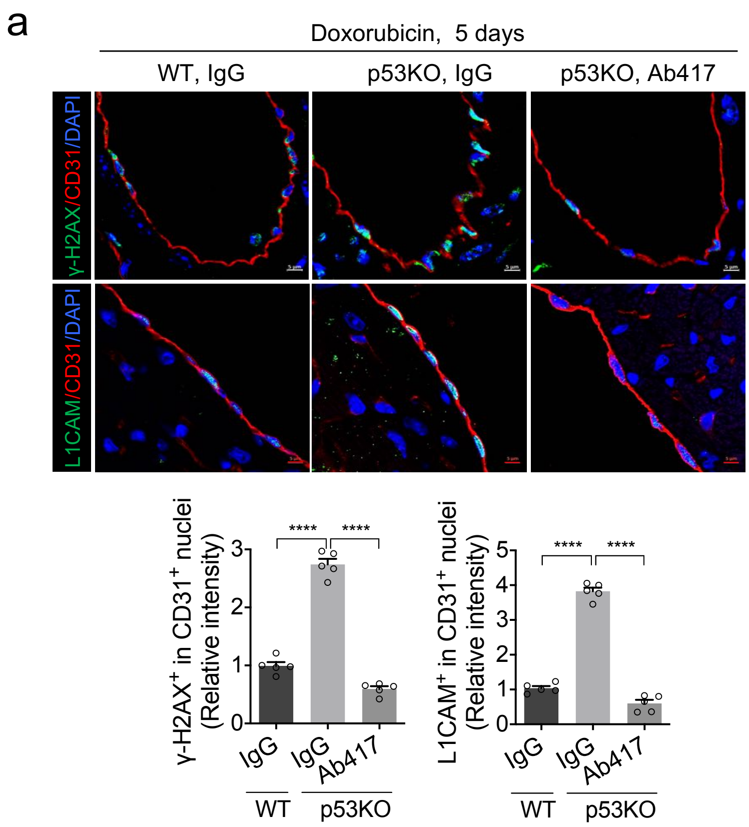

Supplementary Fig. 6 Anti-L1CAM antibody prevents the p53 deficiency-induced increase in L1CAM C-terminal (L1-CT) expression and γ-H2AX foci formation against Dox treatment. (**a**) Immunofluorescence detection of γ-H2AX, L1CAM, and CD31 in heart tissues 5 days after Dox treatment and quantification of γ-H2AX$^+$ cells or nuclear L1CAM$^+$ cells among CD31$^+$ cells (magnification, 400×). Scale bar = 5 μm. Wild-type or EC-p53KO mice were injected intravenously with control IgG or Ab417 (10 mg/kg) 3 times for a week and treated with Dox. Error bars represent mean ±SEM from *n*=5 animals per group (**** $p < 0.0001$, one-way ANOVA).

Supplementary Figure 7

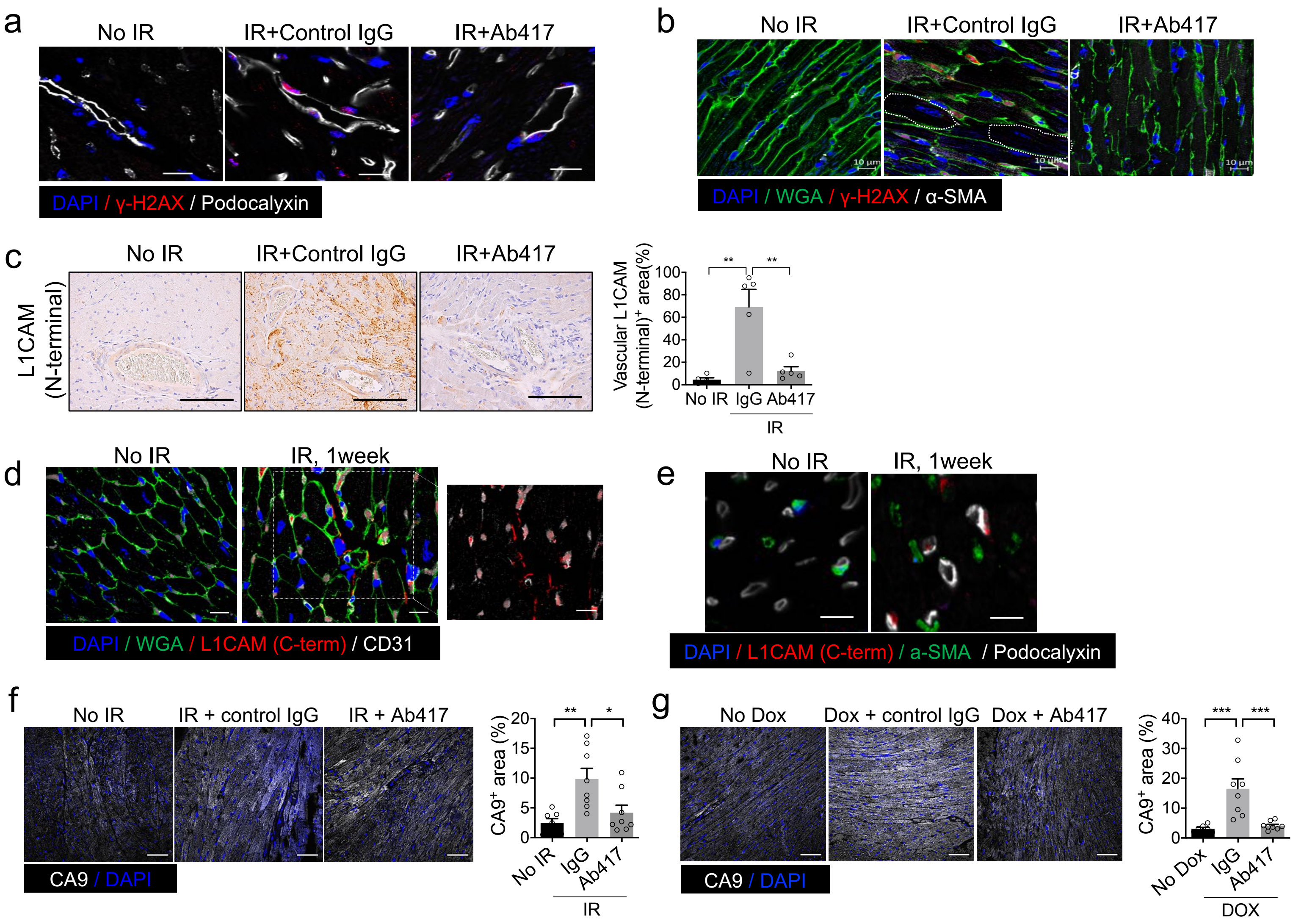

Supplementary Fig. 7 Heart irradiation (IR) induces DNA damage and tissue hypoxia, as well as nuclear L1CAM localization in endothelial cells but not in cardiomyocytes. (a, b) Immunofluorescence detection of WGA, γ-H2AX, α-SMA, and Podocalyxin in heart tissues 1 week post IR. Scale bar = 10 µm. **(c)** Immunohistochemical detection of N-terminal L1CAM in heart tissues 1 week post IR. Mice were injected intravenously with control IgG or Ab417 (10 mg/kg) three times a week (for 1 week) and received 16-Gy whole-heart IR (No IR n=5; IR+control IgG n=5; IR+Ab417 n=5). Quantification of N-terminal L1CAM^{+} area per field (magnification, 200×). Scale bar = 200 µm. Error bars represent mean ±SEM (No IR vs IR+IgG p=0.001; IR+IgG vs IR+Ab417 p=0.028). **(d)** Immunofluorescence detection of WGA, L1-CT, and CD31 in heart tissues 1 week post IR. Scale bar = 10 µm. **(e)** Immunofluorescence detection of WGA, L1-CT, α-SMA, and Podocalyxin in heart tissues 1 week post IR. Scale bar = 10 µm. **(f, g)** Immunofluorescence staining of CA9 (No IR n=7; IR+control IgG n=8; IR+Ab417 n=8, No Dox n=7; Dox+control IgG n=8; Dox+Ab417 n=8) and quantification of CA9^{+} area per field (magnification, 400×). Scale bar = 20 µm. Error represent mean ±SEM (No IR vs IR+IgG p=0.0026; IR+IgG vs IR+Ab417 p=0.0148; No Dox vs Dox+IgG p=0.0003; Dox+IgG vs Dox+Ab417 p=0.0005). **(c,f,g** : one-way ANOVA).

Supplementary Figure 8

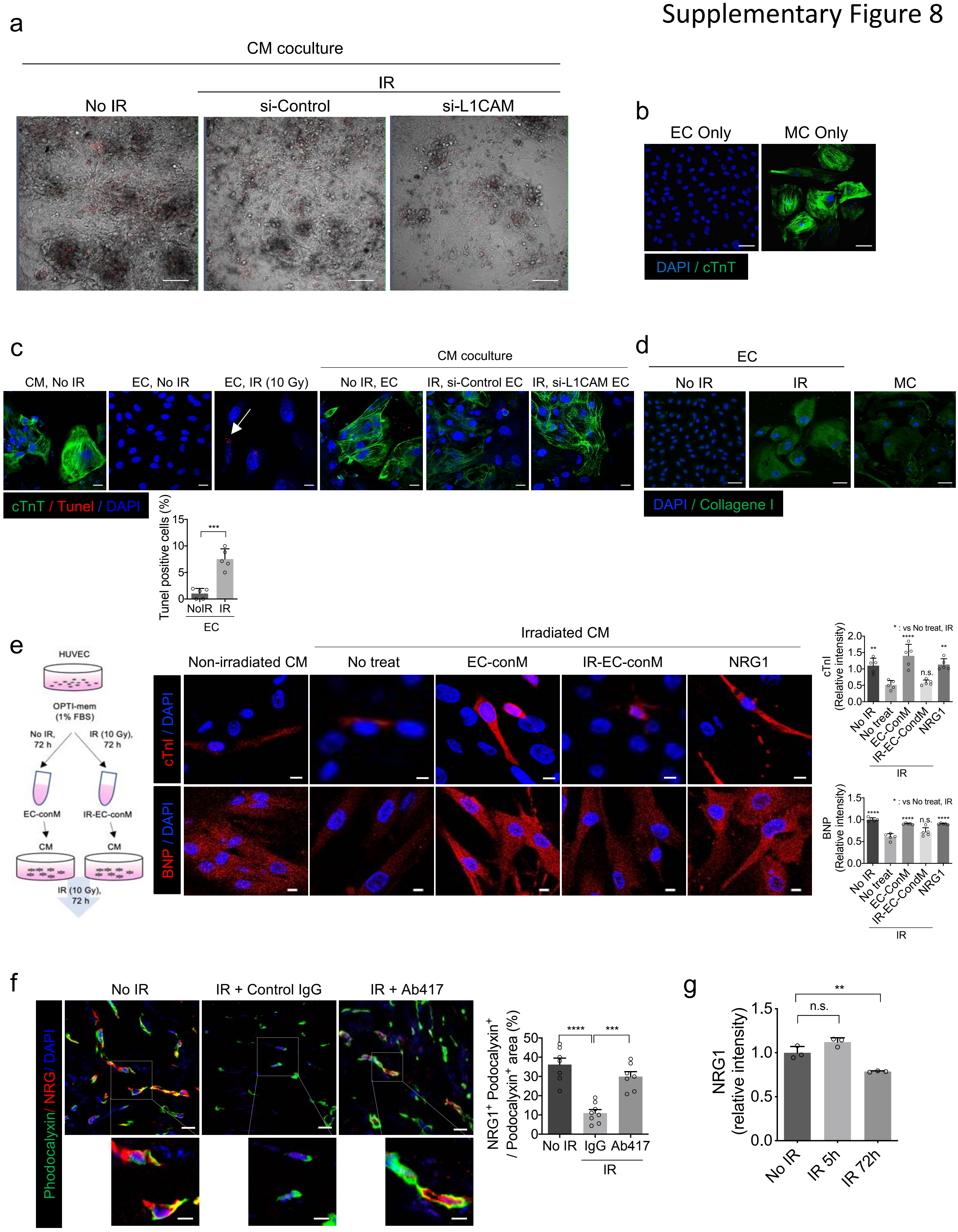

Supplementary Fig. 8 Co-culture of iPSC-CMs and irradiated ECs transfected with control or L1CAM. (a) ECs were transfected with control or L1CAM-specific siRNA and irradiated with 10 Gy. 48 h after initial irradiation (IR); ECs were co-cultured with iPSC-CMs. The representative beating images of the iPSC-CMs were examined after 5 days of co-culture with irradiated ECs (**b, d)** Immunofluorescence staining of cTnT expression in ECs and CMs and collagen I of non-irradiated ECs, irradiated ECs and MCs. Scale bar =50 μm. (magnification, 200×). (**c**) Immunofluorescence staining for cTnT and Tunel in non-irradiated ECs, irradiated ECs, MCs, and co-cultured cells (magnification, 400×). Quantification of Tunel-positive cells in non-irradiated ECs and irradiated ECs Scale bar = 10 μm. Tunel-positive cells percentage were counted at least 20 cells per field (magnification, 200×, n=5). Error bars represent mean ±SEM (p=0.0002). **(e)** Schematic diagram of irradiated CM treatment pretreated with conditioned medium (left). Conditioned medium from HUVECs 72 h after no IR or 10-Gy IR. Immunofluorescence staining for cTnI (top) and BNP (bottom) in CM 72 h post IR (10 Gy) along with the pretreatment of conditioned medium or NRG-1 (30 ng/ml). Scale bar = 10 μm. Quantification of cTnI and BNP intensity per field is shown (magnification, 200×; n=5). Error bars represent mean ±SD (cTnI intensity : NoIR vs IR p=0.0026; IR vs IR+EC-CondM p<0.0001; IR vs IR+NRG1 p=0.0046, BNP intensity : **** p<0.0001). **(f)** Immunofluorescence detection of podocalyxin and NRG-1 in heart tissues 1 week post IR. Mice were injected intravenously with control IgG or Ab417 (10 mg/kg) three times a week (for 1 week) and received 16-Gy whole-heart IR (No IR n=7; IR+control IgG n=8; IR+Ab417 n=8). Scale bar =10 μm (enlarged, 5 μm). Quantification of the $NRG1^+$ area in the $podocalxyin^+$ area. Error bars represent mean ±SEM (No IR vs IR+IgG p < 0.0001; IR+IgG vs IR+Ab417 p=0.0001). **(g)** RNA-seq analysis results showing NRG1 level in HUVECs before and after 10-Gy IR. Total RNA was isolated from HUVECs before and after 10-Gy IR (5 and 72 h). Error bars represent mean ±SD (No IR vs IR 72 h p=0.0048). (**c** : Student's t-test, **e,f,g** : one-way ANOVA for multiple comparisons)

Supplementary Figure 9

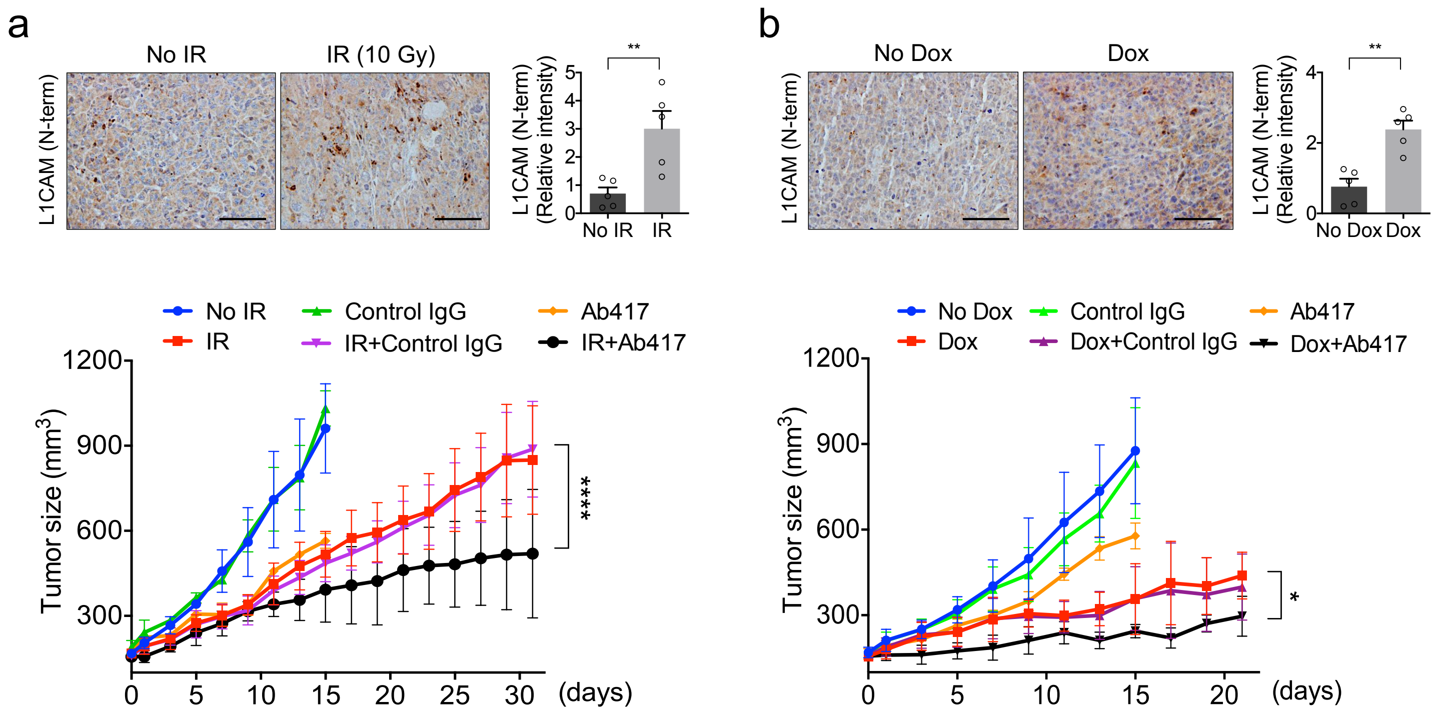

Supplementary Fig. 9 Therapeutic potential of Ab417 in patients with breast cancer receiving radiotherapy and doxorubicin (Dox). (a) MDA-MB-231 tumor-bearing mice were injected intravenously with control IgG or Ab417 (10 mg/kg) three times a week for 2 weeks and received 16-Gy thoracic irradiation (IR). Tumor tissues were obtained from non-irradiated animals (day 0) and 3, 7, 18, and 31 days after IR (n=5 animals per group). Immunohistochemical detection and quantification of N-terminal L1CAM in tumor tissues 18 days after IR (magnification, 400x). Scale bar = 40 µm. Error bars represent mean ±SEM (student's t-test, p=0.0086). Representative images showing tumor growth after IR with or without Ab417 are provided (p>0.0001). **(b)** MDA-MB-231 tumor-bearing mice were injected intravenously with control IgG or Ab417 (10 mg/kg) three times a week for 2 weeks and with 5 mg/kg Dox (once a week). Tumor tissues were obtained from non-Dox animals (day 0) and 21 days after Dox treatment (n=5 animals per group). Immunohistochemical detection and quantification of N-terminal L1CAM in tumor tissues at the indicated time points 21 days after Dox treatment (magnification, 400x). Scale bar = 40 µm. Error bars represent mean ±SEM (Student's t-test, p=0.0013). Representative images showing tumor growth after Dox treatment with or without Ab417 are provided (p=0.0137). For **a** and **b**, error bars of tumor growth graphs indicate represent mean ±SD (one-way ANOVA). Data are representative of three independent experiments.

Supplementary Figure 10

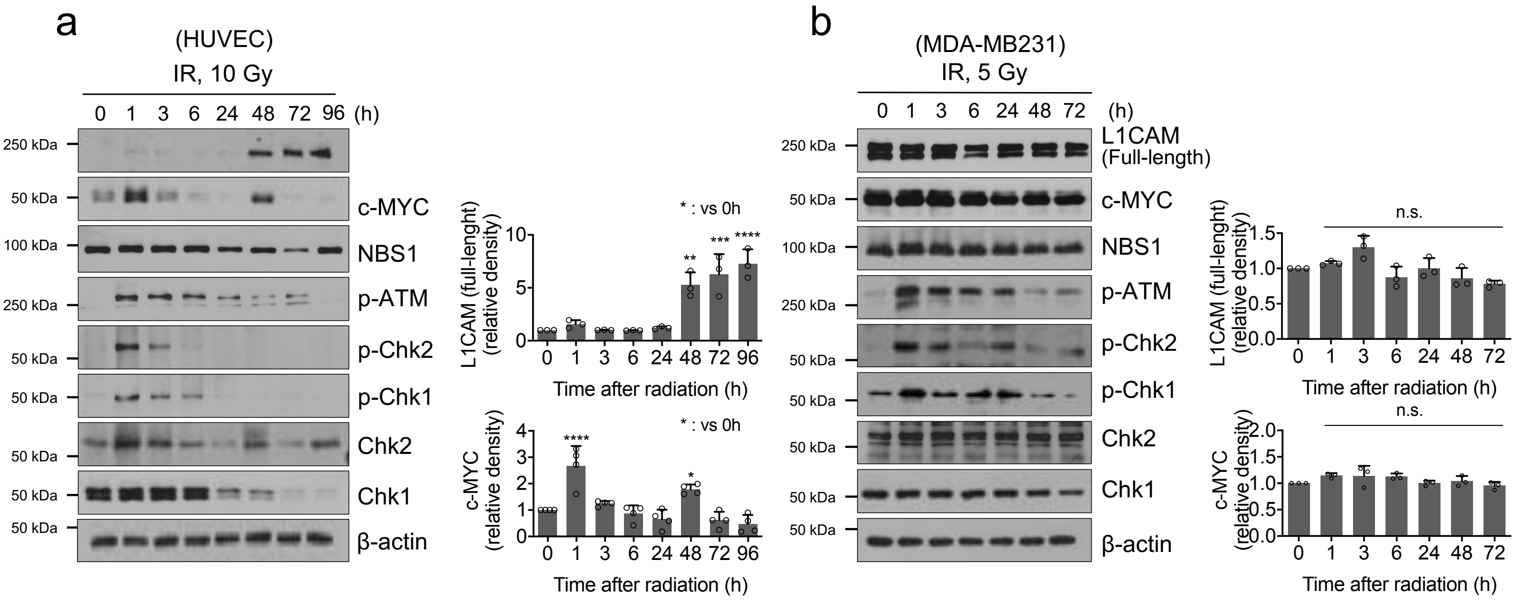

Supplementary Fig. 10 L1CAM expression and DNA damage checkpoint signaling in HUVECs after irradiation (IR). (a, b) Immunoblotting of full-length L1CAM, c-MYC, NBS1, p-ATM, p-Chk2, p-Chk1, Chk2, Chk1 and β-actin in HUVECs and MDA-MB-231 tumor cells at the indicated time points after IR. Quantification of full-length L1CAM and c-MYC is shown. Error bars represent mean ±SD from n=3 independent experiments (HUVEC full-length L1CAM : IR 0h vs 48h p=0.0013; 0h vs 72h p=0.0001; 0h vs 48h p<0.0001, HUVEC c-MYC IR 0h vs 1h p<0.0001; 0h vs 48h p=0.0362, one-way ANOVA).

Supplementary Figure 11

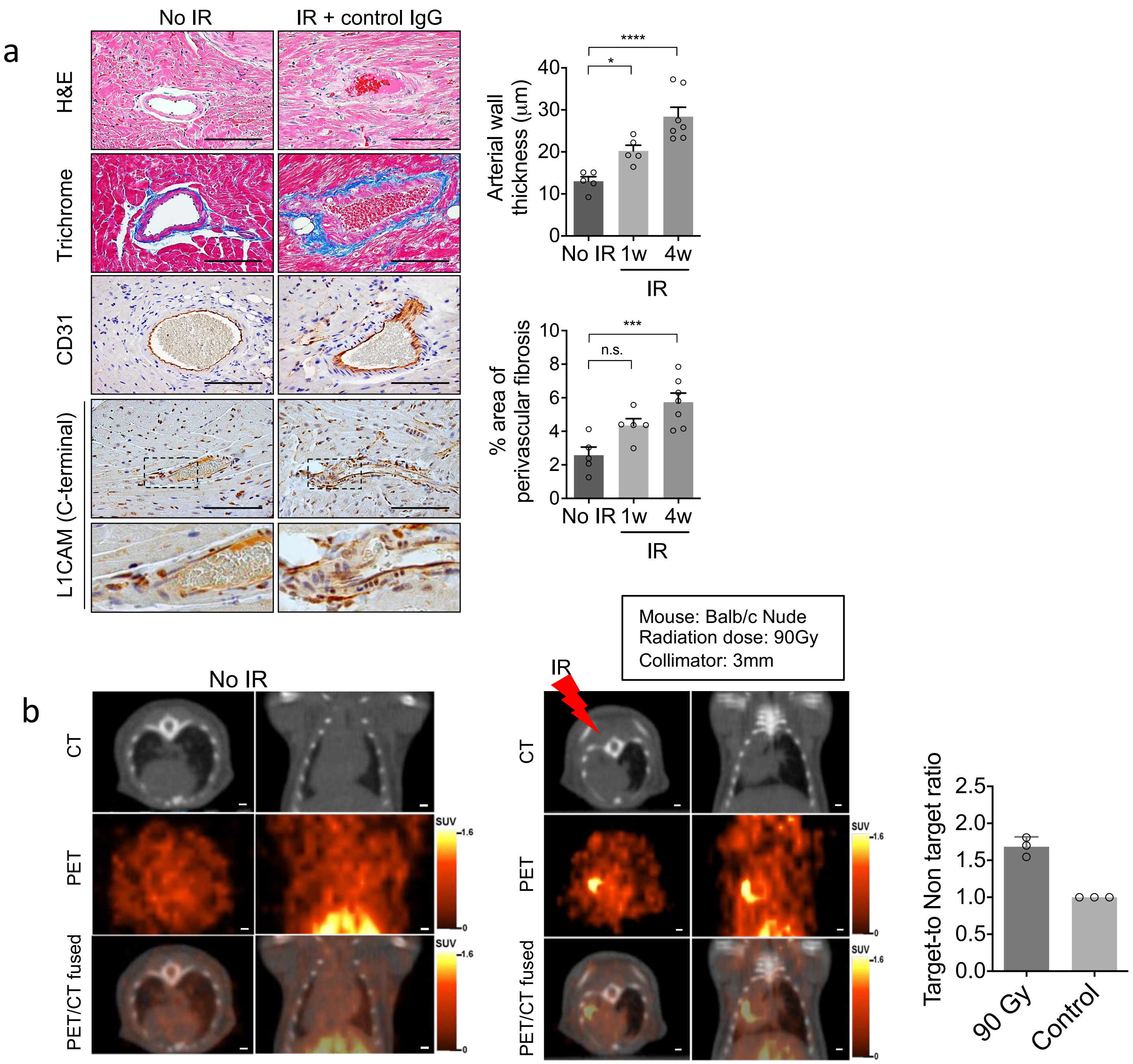

Supplementary Fig. 11 Focal irradiation (IR) causes extensive cardiotoxicity in mice. (a) Hematoxylin and eosin staining, Masson's trichrome staining, and immunohistochemical detection of CD31 L1-CT in cardiac tissue irradiated with 90 Gy using a 3-mm diameter collimator (No IR n=5; IR 1w n=5; IR 4w n=7), and quantification of arterial wall thickness and perivascular fibrosis area per field (magnification, 200×). Scale bar = 100 µm. Error bars represent mean ±SEM (arterial wall thickness : No IR vs IR 1w p=0.0399; No IR vs IR 4w p<0.0001, perivascular fibrosis area : No IR vs IR 4w p=0.001, one-way ANOVA). **(b)** Representative small-animal transverse and coronal positron emission tomography (PET) SUV images of 64Cu-NOTA-Ab417 in mice focally irradiated with 90 Gy using a 3-mm diameter collimator and non-irradiated (control) mice. PET/CT images were acquired 1 week post IR with 64Cu-NOTA-Ab417 (~3.2 MBq/100 mg/head; right). Scale bar = 2 mm. Quantitative analysis of target (irradiated lesion):non-target (normal lesion) ratio (T/N ratio) in SUV images.

Supplementary Figure 12

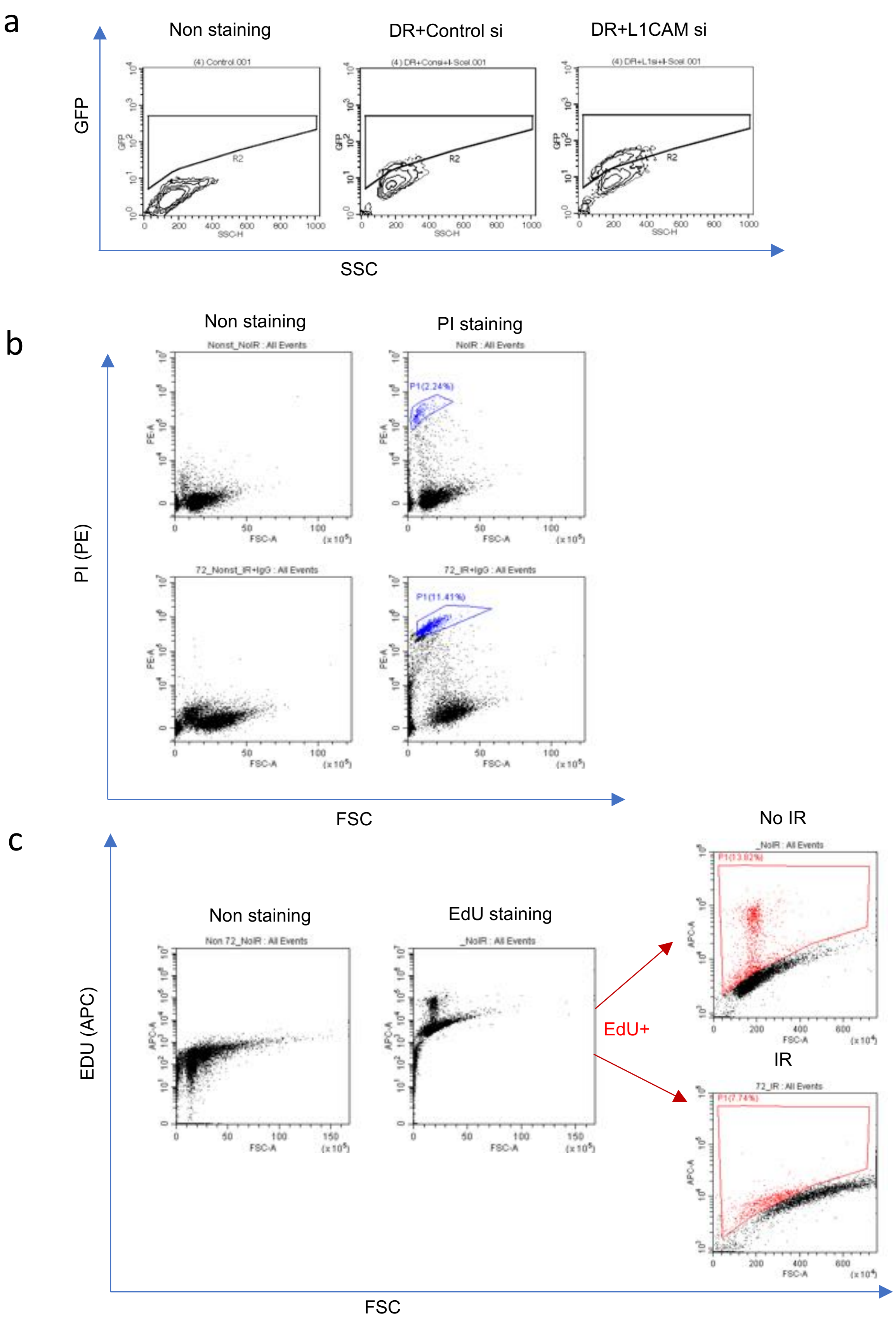

Supplementary Fig 12. Gating strategies for flow cytometry analysis. (a) The rate of GFP-positive cells was defined by non-staining cell populations. **(b)** Cell death rate was identified as propidium iodide-positive cells in Supplementary Fig. 2d, 2h and 5b. **(c)** Proliferative ratio was identified as EdU-positive cells in Supplementary Fig. 2d, 2h and 5b. All experiments were defined by "positive" and "negative" staining cell populations.

Supplementary Table

Supplementary Table 1. Clinicopathologic characteristics of cardiomyopathy patients from whom cardiac tissues were obtained for analysis in this study.

Patient no.	Age	Gender	Sample pathology from pathology verification	Case diagnosis from the donor institution pathology report	Pathology Verification
Normal #1	28	Male	Within normal limits	Within normal limits	Non-Tumor Structures: 100% Myocardium
Normal #2	38	Female	Within normal limits	Within normal limits	Non-Tumor Structures: 5% Endocardium, 95% Myocardium
Normal #3	50	Male	Within normal limits	Within normal limits	Normal great arteries tissue
Normal #4	48	Male	Within normal limits	Within normal limits	Normal cardiac atrium tissue
Normal #5	45	Male	Within normal limits	Within normal limits	Normal myocardial tissue
Normal #6	21	Female	Within normal limits	Within normal limits	Normal auricle of heart tissue
Normal #7	15	Female	Within normal limits	Within normal limits	Normal myocardial tissue
Normal #8	47	Male	Within normal limits	Within normal limits	Normal myocardial tissue
Patient #1	66	Male	Cardiomyopathy	Cardiomyopathy	Myocyte hypertrophy and interstitial fibrosis
Patient #2	61	Male	Cardiomyopathy	Cardiomyopathy	0% endocardium, 80% myocardium with myocyte hypertrophy, 20% epicardium
Patient #3	57	Male	Cardiomyopathy	Cardiomyopathy	100% myocardium
Patient #4	51	Female	Cardiomyopathy	Cardiomyopathy	lesion: 95% abnormal myocardiocytes, 5% scar
Patient #5	46	Female	Cardiomyopathy	Cardiomyopathy	100% myocardium
Patient #6	51	Female	Cardiomyopathy	Cardiomyopathy	Myocardium with diffuse myocyte hypertrophy with focal interstitial fibrosis
Patient #7	64	Female	Myocyte hypertrophy	Myocardial infarction	Lesion (100%): Myocyte hypertrophy 100%; Non-Tumor Structures: 95% Myocardium, 5% Epicardium
Patient #8	29	Female	Myocyte hypertrophy	Leukemia, acute myeloid	Lesion (100%): Myocyte hypertrophy 100%; Non-Tumor Structures: 5% Endocardium, 90% Myocardium, 5% Epicardium
Patient #9	66	Male	Cardiomyopathy	Cardiomyopathy	Focal fibrosis and minimal interstitial lymphocytic infiltrate; Myocyte hypertrophy
Patient #10	51	Female	Cardiomyopathy	Cardiomyopathy	Myocardium with diffuse myocyte hypertrophy with focal interstitial fibrosis
Patient #11	46	Female	Cardiomyopathy	Cardiomyopathy	dilated cardiomyopathy with myocyte hypertrophy and patchy interstitial fibrosis; 0% endocardium, 95% myocardium, 5% epicardium
Patient #12	51	Female	Cardiomyopathy	Cardiomyopathy	lesion: 95% abnormal myocardiocytes, 15% scar; no normal architecture

Supplemental Material

Detailed Methods

Cell culture and monocyte transmigration assay

Primary human coronary artery endothelial cells (HCAECs), isolated from the coronary artery, were purchased from Promocell. HUVECs were plated in 24-well migration plates (containing 24 cell culture inserts; 3 μm pore size) and pretreated with control IgG or Ab417 (20 μg/mL), 1 h before irradiation. Monocytes were fluorescently labeled with CellTracker™ Green CMFDA (C7025; Invitrogen). To the subsequent HUVEC irradiation, CMFDA-labeled monocytes were added and cultured for 24 h. Media from the lower chamber, containing the migrated monocytes, were collected and imaged.

GFP-53BP1 overexpression and live-cell imaging

HUVECs were transfected with a GFP-53BP1 Tagged ORF Clone (Origene, #RG227930) using Lipofectamine 2000 (Invitrogen) according to the manufacturer's instructions. Cells were irradiated with γ-rays (10 Gy). For live-cell time lapse microscopy of the GFP-53BP1 foci, an EVOS M7000 microscope (Invitrogen) equipped with an incubator at 37 °C with humidified 5% CO2, using a ×40 objective lens was used.

Laser microirradiation

Cells were cultured on a 35-mm imaging dish with a glass bottom (200350; SPL). DNA damage was induced via microirradiation of a single region in the nucleus, using a 405 nm laser beam from the LSM700 confocal microscope. Cells were incubated at 37°C for 10 min, fixed, and immunostained as previously described.

Lentiviral vectors and transduction

Plasmid expression vectors encoding full-length L1CAM (phL1A-pcDNA3, Addgene plasmid #12307), nuclear localization sequence-mutated L1CAM (L1-4A, Addgene plasmid #13268) and endocytosis-deficient L1CAM (L1-dRSLE, Addgene plasmid #13266) were purchased encoding human L1CAMs were subcloned into the pLL_EGFP vector. Lentiviral particles were produced in 293T cells by co-transfection with pMDLg_pRRE, pRSV_Rev, and pMD2.G packaging vectors. For transduction, cells were cultured in Opti-MEM (Invitrogen) with EGFP, L1CAM-EGFP, L1-4A-EGFP, or L1-dRSLE-EGFP lentiviral particles.

Cell culture and Treatments

For the lysosomal degradation test, HUVECs were transfected with a L1CAM-WT lentiviral vector using Lipofectamine 2000 (Invitrogen) according to the manufacturer's instructions. HUVECs expressing L1CAM-WT were pretreated with bafilomycin-A1 (100 nM, Sigma, #B1793) for 1 h and incubated for 24 h after Ab417 (40 μg/mL) treatment.

ADCC assay

For ADCC assay, ADCC Reporter Bioassays complete Kit (Promega, #G7015) was used according to the manufacturer's instructions. ADCC Bioassay Target Cells (Raji or HUVECs) were incubated with a series of concentrations of Ab, anti-CD20, and Ab417, followed by the addition of ADCC Bioassay Effector Cells. The E:T ratio was 6:1. After 6 h of induction at 37 °C, Bio-Glo™ Luciferase Assay Reagent was added and incubated at ambient temperature for 15 min. Luminescence was determined using a Luminometer (Spectramax i3, Molecular Devices). For ADCC Reporter Bioassay, the starting concentrations and serial dilution schemes are 1×10^{-6}g/ml, 3-fold serial dilution for anti-CD20 and Ab417.

Antibodies for immunoblotting and immunohistochemistry

Immunoblotting and immunofluorescence staining were performed using primary antibodies against p-CHK1 (immunoblotting 1:1000; #2344; Cell Signaling), CHK-1 (immunofluorescence 1:1000; #2360; Cell Signaling), p-CHK2 (immunoblotting 1:1000; #2661; Cell Signaling), CHK2 (immunoblotting

1:1000; #2662; Cell Signaling), p-ATM (immunoblotting 1:5000; #05–740; Millipore), ATM (immunoblotting 1:5000; #05–513; Millipore), NBS1 (immunoblotting 1:1000; sc-515069; Santa Cruz Biotechnology), Podocalyxin (immunofluorescence 1:200; AF1556; R&D Systems), cTnT (immunofluorescence 1:200; MAB18742; R&D Systems), cTnI (immunofluorescence 1:200; ab47003; Abcam), BNP (immunofluorescence 1:200; sc-271185; Santa Cruz Biotechnology), CA-9 (immunofluorescence 1:200; NB100-147; NOVUS), WGA (immunofluorescence 1:500; #W11261; Invitrogen), cTnT (immunofluorescence 1:200; #MAB18742; R&D Systems).

Treatment Conditioned medium from ECs on CMs

Human primary adult cardiomyocytes (CM)s were purchased from Sciencell (#6200) and cultured in 50 μg/mL fibronectin-coated dishes, according to the manufacturer's protocols. HUVECs were incubated in Opti-MEM (1% FBS, Thermo Fisher Scientific) for 72 h after non-irradiation or irradiation to produce the conditioned medium. Before CMs were irradiated, the CM medium was modified with conditioned medium or pretreated with NRG-1 (30ng/ml, R&D system, #5898-NR). 72 hours after irradiation, the CMs were fixed in 10% (v/v) neutral-buffered formalin and subjected to TnI and BNP staining for immunofluorescence analysis.

Syngeneic tumor models

MDA-MB-231 cells (4×10^6 cells) were injected into the mammary fat pad of female nude mice. Tumor volumes were determined according to the formula (L × W × H)/2, using the caliper-measured tumor length (L), width (W), and height (H). When tumor volumes reached 150–200 mm^3, thoracic irradiation was performed using the X-RAD 320 platform (Precision X-ray) or intraperitoneal Dox injection (5 mg/kg, 3 times/3 weeks). Ab417 (10 mg/kg, 6 times/2 weeks) was injected intravenously, starting 1 h before Dox or radiation treatment.
