# Supplementary material for: An antibody against L1 cell adhesion molecule inhibits cardiotoxicity by regulating persistent DNA damage
Source: Nat Commun. 2021 Jun 2;12:3279. doi: 10.1038/s41467-021-23478-1 (PMC8172563; doi:10.1038/s41467-021-23478-1)
Supplement: Supplementary file 3 — Description of Additional Supplementary Files [file 41467_2021_23478_MOESM3_ESM.docx]

Description of Additional Supplementary Files

Title: Supplementary Movie 1

Description: Fig.S8a, Co-culture of iPSC-CMs and non-irradiated ECs

ECs were transfected and irradiated with 10 Gy. 48 h after initial irradiation (IR); ECs were co-cultured with iPSC-CMs. The representative beating movie of the iPSC-CMs was examined after 5 days of co-culture with non-irradiated ECs.

Title: Supplementary Movie 2

Description: Fig.S8a, Co-culture of iPSC-CMs and irradiated ECs transfected with control siRNA

ECs were transfected with control siRNA and irradiated with 10 Gy. 48 h after initial irradiation (IR); ECs were co-cultured with iPSC-CMs. The representative beating movie of the iPSC-CMs was examined after 5 days of co-culture with irradiated ECs transfected with control siRNA.

Title: Supplementary Movie 3

Description: Fig.S8a, Co-culture of iPSC-CMs and irradiated ECs transfected with L1CAM siRNA

ECs were transfected with L1CAM-specific siRNA and irradiated with 10 Gy. 48 h after initial irradiation (IR); ECs were co-cultured with iPSC-CMs. The representative beating movie of the iPSC-CMs was examined after 5 days of co-culture with irradiated ECs transfected with L1CAM siRNA.
