# Supplementary material for: An antibody against L1 cell adhesion molecule inhibits cardiotoxicity by regulating persistent DNA damage
Source: Nat Commun. 2021 Jun 2;12:3279. doi: 10.1038/s41467-021-23478-1 (PMC8172563; doi:10.1038/s41467-021-23478-1)
Supplement: Supplementary file 7 — Reporting Summary [file 41467_2021_23478_MOESM7_ESM.pdf]

nature research

Corresponding author(s): DBPR, NCOMM_20_30626A

Last updated by author(s): Mar 27, 2021

Reporting Summary

Nature Research wishes to improve the reproducibility of the work that we publish. This form provides structure for consistency and transparency in reporting. For further information on Nature Research policies, see our Editorial Policies and the Editorial Policy Checklist.

Statistics

For all statistical analyses, confirm that the following items are present in the figure legend, table legend, main text, or Methods section.

n/a	Confirmed	
☐	☒	The exact sample size (*n*) for each experimental group/condition, given as a discrete number and unit of measurement
☐	☒	A statement on whether measurements were taken from distinct samples or whether the same sample was measured repeatedly
☐	☒	The statistical test(s) used AND whether they are one- or two-sided<br>*Only common tests should be described solely by name; describe more complex techniques in the Methods section.*
☒	☐	A description of all covariates tested
☒	☐	A description of any assumptions or corrections, such as tests of normality and adjustment for multiple comparisons
☐	☒	A full description of the statistical parameters including central tendency (e.g. means) or other basic estimates (e.g. regression coefficient) AND variation (e.g. standard deviation) or associated estimates of uncertainty (e.g. confidence intervals)
☐	☒	For null hypothesis testing, the test statistic (e.g. *F*, *t*, *r*) with confidence intervals, effect sizes, degrees of freedom and *P* value noted<br>*Give P values as exact values whenever suitable.*
☒	☐	For Bayesian analysis, information on the choice of priors and Markov chain Monte Carlo settings
☒	☐	For hierarchical and complex designs, identification of the appropriate level for tests and full reporting of outcomes
☐	☒	Estimates of effect sizes (e.g. Cohen's *d*, Pearson's *r*), indicating how they were calculated

Our web collection on statistics for biologists contains articles on many of the points above.

Software and code

Policy information about availability of computer code

Data collection	We used LSM 880, FACSCalibur and FACSAria cell sorter (BD Biosciences) for collecting images and flow cytometry data.
Data analysis	We used Prism 7 (Graphpad), Image J 1.49v (NIH), R 1.0.12, Zen 3.2 (Zeiss) and Diva 7.0 (BD Biosciences) for data and image analysis and plotting.

For manuscripts utilizing custom algorithms or software that are central to the research but not yet described in published literature, software must be made available to editors and reviewers. We strongly encourage code deposition in a community repository (e.g. GitHub). See the Nature Research guidelines for submitting code & software for further information.

Data

Policy information about availability of data

All manuscripts must include a data availability statement. This statement should provide the following information, where applicable:
- Accession codes, unique identifiers, or web links for publicly available datasets
- A list of figures that have associated raw data
- A description of any restrictions on data availability

The RNA-seq data was deposited in Gene Expression Omnibus (GEO) with accession number GSE155285 [https://www.ncbi.nlm.nih.gov/geo/query/acc.cgi?acc=GSE155285] Source data are provided with this paper. All other relevant data are available from the corresponding author upon reasonable request. We put this information into the 'Data availability' section explicitly

Field-specific reporting

Please select the one below that is the best fit for your research. If you are not sure, read the appropriate sections before making your selection.

- [x] Life sciences
- [ ] Behavioural & social sciences
- [ ] Ecological, evolutionary & environmental sciences

For a reference copy of the document with all sections, see nature.com/documents/nr-reporting-summary-flat.pdf

Life sciences study design

All studies must disclose on these points even when the disclosure is negative.

Sample size	Tumor volumes were determined according to the formula (L × W × H)/2, using the caliper-measured tumor length (L), width (W), and height (H).
Data exclusions	No data were excluded.
Replication	All attempts at replication were successful.
Randomization	Animals were randomly divided into experimental groups.
Blinding	Mice were randomly assigned to each experimental group.

Reporting for specific materials, systems and methods

We require information from authors about some types of materials, experimental systems and methods used in many studies. Here, indicate whether each material, system or method listed is relevant to your study. If you are not sure if a list item applies to your research, read the appropriate section before selecting a response.

Materials & experimental systems

n/a	Involved in the study	
	x	Antibodies
	x	Eukaryotic cell lines
x		Palaeontology and archaeology
	x	Animals and other organisms
x		Human research participants
x		Clinical data
x		Dual use research of concern

Methods

n/a	Involved in the study	
x		ChIP-seq
	x	Flow cytometry
x		MRI-based neuroimaging

Antibodies

Antibodies used	Immunoblotting, immunohistochemistry, and immunofluorescence staining were performed using primary antibodies against the N-terminal domain of L1CAM (immunohistochemistry/immunofluorescence 1:100; sc-31032; Santa Cruz Biotechnology), L1-CT (immunohistochemistry/immunofluorescence 1:500, immunoblotting 1:1000; LS-B9803; LSBio; immunofluorescence 1:200; sc-53386; Santa Cruz Biotechnology; immunofluorescence 1:200; ab123990; Abcam), complete L1CAM (immunoblotting 1:1000; sc-53386; Santa Cruz Biotechnology), γ-H2AX (immunohistochemistry/immunofluorescence 1:500, immunoblotting 1:1000; 05-636; Millipore), CD31 (immunohistochemistry/immunofluorescence 1:200; #AF3628; R&D Systems; immunoblotting 1:1000; 28364; Abcam), GAPDH (immunoblotting 1:1000; sc-47724; Santa Cruz Biotechnology), lamin B (immunoblotting 1:1000; sc-6216; Santa Cruz Biotechnology), αSMA (1:2000; A5228; Sigma-Aldrich), VCAM-1 (immunoblotting 1:1000; sc-1504; Santa Cruz Biotechnology), p-ATM (immunofluorescence 1:200; 05-740; Millipore), 53BP1 (immunofluorescence 1:200; sc-10914; Santa Cruz Biotechnology), and DNA-PKcs (immunofluorescence 1:200; sc-135886; Santa Cruz Biotechnology). To visualize actin stress fibres, cells were stained with Alexa Fluor 488-conjugated phalloidin (1:40; Invitrogen)
Validation	All antibodies were used under manufacturer's recommendations. The dilution ratios are mentioned in methods.

Eukaryotic cell lines

Policy information about cell lines

Cell line source(s)	We described in the Methods section. HUVECs (#C-12203)and human CD14+ monocytes (C#12909) were obtained from PromoCell and cultured in Endothelial Cell Growth Medium 2 and Mononuclear Cell Medium (PromoCell).iPSC-CMs were purchased from Cellartis (#Y50025) and cultured in 50 µg/mL fibronectin-coated dishes according to the manufacturer's protocols. Human primary adult cardiomyocytes (CM)s were purchased from Sciencell (#6200) and cultured in 50 µg/mL fibronectin-coated dishes, according to the manufacturer's protocols

Authentication	The catalog number and manufacturer info. have been porvided in the " Method" section. The validation of cell line are described in the manufacturer's web-page.
Mycoplasma contamination	Confirmed to be free of contamination.
Commonly misidentified lines (See ICLAC register)	No commonly misidentified lines were used in the study

Animals and other organisms

Policy information about studies involving animals; ARRIVE guidelines recommended for reporting animal research

Laboratory animals	All animal experiments were approved by the Institutional Animal Care and Use Committee of the Korea Institute of Radiological & Medical Sciences and are reported in accordance with the Animal Research: Reporting of In Vivo Experiments (ARRIVE) guidelines. Specific pathogen-free female BALB/c nude mice were purchased from Orient Bio. Specific pathogen-free C57BL/6 Tie2-Cre and Trp53flox/flox mice were purchased from the Jackson Laboratory. Male Tie2-Cre and Tie2-Cre;Trp53flox/+ mice were crossed with female Trp53flox/flox mice to generate Tie2-Cre;Trp53flox/+ and Tie2-Cre;Trp53flox/flox mice, respectively. Tie2-Cre;Trp53+/+ or Tie2-Cre–/– littermates were used as controls. All animal experimental data provided are representative of three independent experiments. All experiments were conducted with 6–8-week-old mice, and the mice were maintained on a 12 h light-dark cycle in a standard environment (20 ± 1 °C room temperature, 50 ± 10 % relative humidity) with a standard diet and water ad libitum. All mice were anaesthetized with a combination of anaesthetics before being euthanized.
Wild animals	The study did not involve wild animals.
Field-collected samples	the study did not involve samples collected from the field.
Ethics oversight	All animal experiments were approved by the Institutional Animal Care and Use Committee of the Korea Institute of Radiological & Medical Sciences and are reported in accordance with the Animal Research: Reporting of In Vivo Experiments (ARRIVE) guidelines.

Note that full information on the approval of the study protocol must also be provided in the manuscript.

Flow Cytometry

Plots

Confirm that:

- [x] The axis labels state the marker and fluorochrome used (e.g. CD4-FITC).
- [x] The axis scales are clearly visible. Include numbers along axes only for bottom left plot of group (a 'group' is an analysis of identical markers).
- [x] All plots are contour plots with outliers or pseudocolor plots.
- [x] A numerical value for number of cells or percentage (with statistics) is provided.

Methodology

Sample preparation	pHPRT-DRGFP or pimEJ5GFP was transfected into HUVECs along with control or L1CAM-targeting siRNA using Lipofectamine 2000 reagent (Invitrogen). After 1 day, the cells were transfected with pBAD-I-SceI to induce DNA damage and cultured for 48 h. The cells positive for GFP because of DNA repair were analysed on a FACSCalibur flow cytometer (Becton Dickinson, Franklin Lakes, NJ) to estimate the frequencies of HR and NHEJ.
Instrument	FACSCalibur and FACSAria cell sorter (BD biosciences)
Software	FACS DIVA software (BD biosciences)
Cell population abundance	Cells were not sorted in the current study.
Gating strategy	For the gating strategy, all experiments are defined by "positive" and "negative" staining cell populations

- [x] Tick this box to confirm that a figure exemplifying the gating strategy is provided in the Supplementary Information.
